# Investigation of Transforming Growth Factor-β1 Gene Polymorphisms Among Iranian Patients With Chronic Hepatitis C

**DOI:** 10.5812/kowsar.1735143X.776

**Published:** 2011-11-20

**Authors:** Sara Romani, Pedram Azimzadeh, Seyed Reza Mohebbi, Shabnam Kazemian, Shohreh Almasi, Hamed Naghoosi, Faramarz Derakhshan, Mohammad Reza Zali

**Affiliations:** 1 Research Center for Gastroenterology and Liver Diseases, Shahid Beheshti University of Medical Sciences, Tehran, IR Iran

**Keywords:** Iranian Hepatitis C, chronic Transforming Growth Factor Beta1 Polymorphism

## Abstract

**Background:**

Chronic hepatitis C infection is caused by the hepatitis C virus (HCV), and its clinical complications include liver cirrhosis, liver failure, and hepatocellular carcinoma.Transforming growth factor-β1 (TGF-β1) is an important cytokine in cell growthand differentiation, angiogenesis, extracellular matrix formation, immune responseregulation, and cancer development and progression.

**Objectives:**

The aim of this study was to investigate the relationship between single nucleotide polymorphisms (SNPs) in TGF-β1 and chronic HCV infection among patients referred to the Taleghani Hospital, Tehran, Iran between 2008 and 2010.

**Patients and Methods:**

In this case-control study, samples were collected using a convenience sampling method. We genotyped 164 HCV patients and 169 healthy controls for 3 SNPs in the TGF-β1 gene (-509 promoter, codon 10, and codon 25). We determined the SNP genotypes by using polymerase chain reaction-restriction fragment length polymorphism (PCR-RFLP) method. To confirm the PCR-RFLP genotyping results, 10% of the samples were re-genotyped using a direct sequencing method.

**Results:**

There were no significant differences in the allelic frequency distribution of SNPs at -509 C/T, +869 C/T, or +915 G/C between HCV patients and the healthy controls. Genotyping results for all three polymorphic sites were similar with no statistically significant differences between the groups.

**Conclusions:**

Most of the Iranian patients (over 85%), both healthy controls and HCV patients, had the GG genotype at the +915 G/C position, resulting in a high level of TGF-β1 production. Therefore, we concluded that the SNPs investigated by us cannot be considered as prognostic factors for HCV infection in our population, despite being reported as prognostic markers in other populations. Moreover, there is a possibility that most of the population is susceptible to HCV infection.

## 1. Background

Approximately 175 million people worldwide are infected with hepatitis C virus (HCV), and about 80% of these individuals develop chronic HCV infection. Some individuals with persistent HCV infections develop liver cirrhosis, which can lead to hepatocellular carcinoma in some patients [1][2][3][4]. Hepatitis C has a complex etiology; factors such as genetic predisposition and cytokine production levels contribute to the pathogenesis [5]. Cytokines play a vital role in the body's ability to fight viral infections: they determine the main type of immune response triggered and directly inhibit viral proliferation [6]. Host genetic variations such as single nucleotide polymorphisms (SNPs) in cytokine genes can affect the rate of HCV production or secretion [7]. Transforming growth factor-β (TGF-β) is an important cytokine for cell growth and differentiation, angiogenesis, extracellular matrix formation, immune response regulation, and cancer development and progression [8][9][10]. Changes in the secretion of immune suppressive factors such as TGF-β can cause dysregulation of host immune response in chronic HCV patients [5][11].The TGF-β superfamily consists of 3 main isoforms, namely, TGf-β1, TGf-β2, and TGf-β3. TGF-β1 is a dimeric protein that acts either as a hormone or locally as a regulator of proliferation, differentiation, extracellular matrix production, and cell death [12]. Characteristics of the extracellular matrix may also contribute to the clinical symptoms of liver diseases such as HCV infection [13]. Several studies have reported differences in cytokine production among patients; this difference could be attributable to variation in the levels of gene expression or in secretion levels of cytokines.

## 2. Objectives

In this study, we investigated three important SNPs in the TGF-β1 gene. Two SNPs are found in the signal sequence of the early producing protein and one in the promoter region. These polymorphisms can affect the gene expression or secretion levels of the mature protein [14][15]. One SNP in codon 25 of TGF-β1's signal sequence was previously associated with an individual's susceptibility to chronic HCV infection [3]. The aim of the present study was to determine whether any of the three selected TGF-β1 polymorphisms are associated with chronic HCV infection in an Iranian population.

## 3. Patients and Methods

### 3.1. Study Population

We conducted a case-control study by using samples collected using a convenience sampling method from individuals at the Taleghani Hospital of Tehran between March 2008 and October 2010. The study population consisted of 333 Iranian individuals, including 164 patients with HCV infection and 169 healthy controls. In the patient group, HCV infection was diagnosed using a third-generation enzyme-linked immunosorbent assay with anti-HCV antibodies (DRG International Inc., USA). We excluded patients co-infected with hepatitis B virus (HBV) or human immunodeficiency virus (HIV); these patients were identified using tests for hepatitis B surface antigen and/or anti-HIV antibodies (DRG International Inc., USA). To confirm HCV infection in patients who yielded positive results in the anti-HCV antibody test, circulating viral RNA was identified using reverse transcription-polymerase chain reaction (RT-PCR). The healthy control group consisted of volunteer blood donors who had no history of hepatitis or hepatobiliary disorders and showed negative results for anti-HCV antibodies and HCV-RNA.

### 3.2. RNA Extraction and RT-PCR

Viral RNA was extracted from 200 µL of plasma with the QIAmp viral RNA mini kit (Qiagen, Hilden, Germany), according to the manufacturer's instructions. Complementary DNA was synthesized in a total volume of 32.5 μL, consisting of 1 μL of random hexamer primers, 5 μL of ssRNA template (100 ng), 4 μL of 5× buffer, 0.5 μL of Ribolock RNase inhibitor, 2 μL of dNTP mix, 200 U of RevertAidTM M-MuLV reverse transcriptase, and 19 μL of RNase-free water (Fermentas, Latvia). Reverse transcription was carried out at 42°C for 1 h.

### 3.3. TGF-β Gene Polymorphisms

Peripheral blood samples from all 333 subjects were collected and stored at 4°C. Genomic DNA was extracted from whole blood by using a standard phenol-chloroform method. Genotyping of the 3 SNPs in the TGF-β gene, namely, the -509 C/T SNP in the promoter, the +869 T/C SNP in codon 10, and the +915 G/C SNP in codon 25, was performed using PCR-restriction fragment length polymorphism (RFLP). The PCR conditions for the SNPs in codons 10 and 25 were as follows: 95°C for 5 min; 35 cycles of 95°C for 30 s, 58.4°C for 30 s, and 72°C for 30 s; and a final extension at 72°C for 10 min. The PCR conditions for the -509 SNP were as follows: 95°C for 5 min; 30 cycles of 95°C for 30 s, 62°C for 45 s, and 72°C for 30 s; and a final extension at 72°C for 10 min. For genotyping the SNP at -509, we used previously described primers [16]. The target sequence was amplified by PCR to yield a 153-bp product. Subsequently, the PCR product was digested using the Eco81I restriction enzyme (Fermentas, Latvia).For genotyping the SNPs of codons 10 and 25, we designed a pair of primers that amplified both polymorphic sites. This PCR product was used as a template for two RFLP reactions with MspA1I (Promega, USA) for codon 10 and BglI (Fermentas, Latvia) for codon 25. The primer sequences, digestion conditions, and restriction enzymes for the detection of each SNP are given in Table 1. Amplification of 100 ng of template DNA for each of the 2 PCR reactions was performed using a total volume of 25 µL containing 10 pmol of forward and reverse primers, 1.25 U of Taq polymerase, 200 µM of each dNTP (Fermentas, Latvia), 37 mM of MgCl2, 1.25 µL of dimethylsulphoxide (Sigma Aldrich, Germany), and distilled deionized water. The PCR products were run on a 1.5% agarose gel and stained with ethidium bromide for visualization under UV light. The RFLP products for codon 25 and -509 promoter SNPs were visualized on a 2% agarose gel; the RFLP products for codon 10 were separated and visualized using 16% polyacrylamide gel electrophoresis.

**Table 1:** Primer Sequence and Restriction Enzymes for Three Polymorphism Sites of TGF-β1 Gene

	**Primer Sequence**	**PCR Annealing Temperature,°C**	**Restriction Enzyme**	**RFLP Incubation Temperature,°C**
Codon 10 (+869)	F: 5- GTTATTTCCGTGGGATACTGAGAC-3	58.4	MspA1I	30
Codon 25 (+915)	R: 5- GACCTCCTTGGCGTAGTAGTCG -3	58.4	BglI	37
Promoter (-509)	F: 5- CAGTAAATGTATGGGGTCGCAG -3	60.2	Eco81I	37
	R: 5- GGTGTCAGTGGGAGGAGGG -3			

### 3.4. DNA Sequencing

To confirm the RFLP genotyping results, we re-genotyped over 10% of the samples (45 samples) by using direct sequencing with an ABI genetic analyzer 3130xl and a chain-termination protocol.

### 3.5. Statistical Analysis

The consistency of the genotyping results was examined with Hardy-Weinberg equilibrium. Logistic regression and chi-square tests were used to compare the genotype frequencies between the HCV and control groups. Age differences between the 2 groups were compared as a quantitative variable by using an independent sample t test. SPSS version 13 software and the logistic regression test were used to evaluate the odds ratio (confidence interval = 95%).

## 4. Results

**Figure 1:**
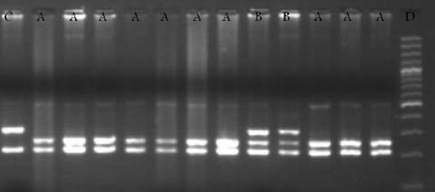
Representative Photograph of RFLP Electrophoresis on 2% Agarose Gel for TGF-β1 Codon 25 Restricted Fragments With BglI Enzyme . A) GG genotype 212bp and 252bp, B) GC genotype 212bp, 252bp and 312bp, C) CC genotype 212bp and 312bp, D) DNA size marker 100bp.

**Figure 2:**
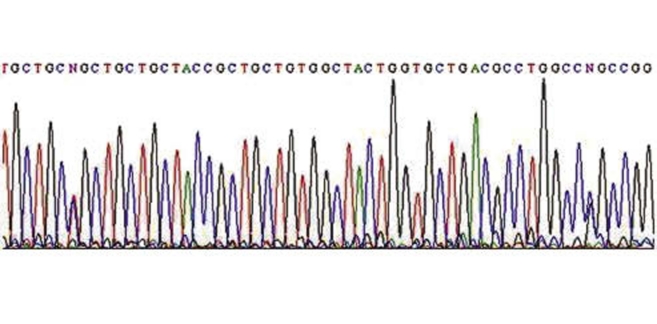
DNA Sequence of TGF-β1 Gene. Codon 10 and Codon 25 polymorphisms. The sequence was read in reverse direction and N shows the site of polymorphism.

In this study, we recruited a total of 164 HCV patients (132 men and 32 women) with a mean age of 47.9 ± 13.8 years. The control group consisted of 169 individuals (85 men and 84 women) with a mean age of 41.07 ± 17.5 years. The percentage of men in the HCV group was higher than that in the control group, and the mean age was higher in the HCV group than in the control group. The TGF-β1 polymorphisms in the -509, +869 (CTG to CCG), and +915 (CGG to CCG) sites were genotyped in all the subjects. Figure 1 shows the RFLP products for codon 25 on a 2% agarose gel. The allelic frequencies of the three SNPs in the chronic HCV patients and the healthy subjects are given in Table 2. The chi-square test was used to analyze the relationship between the genotypes and hepatitis status. There was no significant difference in the allelic distribution of the three polymorphic sites between the HCV group and the control group (P > 0.05).Table 3 presents a summary of the TGF-β1 genotypes for the three polymorphic sites in the promoter, codon 10, and codon 25 among the HCV patients and the healthy controls. According to results of the chi-square test, there were no statistically significant differences in genotype variants of the studied polymorphic sites between the HCV and control groups (P > 0.05). The mean age of the HCV patients was greater than that of the healthy controls, and the percentage of men was higher in the HCV group than in the healthy control group; therefore, we used the logistic regression method to remove these confounding variables. The results of RFLP genotyping for the three polymorphic sites were confirmed using the direct sequencing method Table 4; this showed that the results of the two methods were consistent.Figure 2 shows a sample of the polymorphic sequences for codons 10 and 25.

**Table 2:** Allelic Frequencies of TGF-β Gene Among Patients With Chronic HCV Infection and Healthy Blood Donors

	**Allele**	**Chronic HCV Patients, No. (%)**	**Healthy Blood Controls, No. (%)**	**P value a**
TGF-β codon 10				0.424
	T	183 (54.1)	181 (55.2)	
	C	155 (45.9)	147 (44.8)	
TGF-β codon 25				0.526
	G	308 (93.9)	318 (94.1)	
	C	20 (6.1)	20 (5.9)	
TGF-β -509				0.368
	T	178 (54.3)	178 (52.7)	
	C	150 (45.7)	160 (47.3)	

**Table 3:** Genotypic Frequencies of TGF-β Gene Among Patients With Chronic HCV Infection and Healthy Blood Donors

**Polymorphism**	**Genotype**	**Chronic HCV Patients, No.(%) (n = 164)**	**Healthy Blood Controls, No.(%)(n = 169)**	**P value**
TGF-β codon 10				0.956
	T/T	49 (29)	50 (30.5)	
	C/T	85 (50.3)	81 (49.4)	
	C/C	35 (20.7)	33 (20.1)	
TGF-β codon 25				0.780
	G/G	145 (88.4)	151 (89.3)	
	G/C	18 (11)	16 (9.5)	
	C/C	1 (0.6)	2 (1.2)	
TGF-β -509 (Promoter)				0.920

**Table 4:** Restriction Fragments for Three RFLP Reactions

**TGF-β1 Polymorphism**	**Restriction Enzyme**	**Genotype**	**Restricted Fragments, bp**
Codon 10 (+869)	MspA1I		
		CC	12, 40, 67, 108, 230
		CT	12, 40, 67, 108, 230, 242
		TT	40, 67, 108, 242
Codon 25 (+915)	BglI		
		GG	212, 252, 60
		GC	212, 252, 312
		CC	212, 312
Promoter (-509)	Eco81I		
		TT	153
		CT	153,117,36
		CC	117,36

## 5. Discussion

A number of genes contribute to the difference in the cytokine levels observed in individuals; however, the mechanism of function of these genes has not been fully understood [7][17][18]. Because cytokines regulate the immune response, polymorphisms in cytokine genes or variations in their expression may affect an individual's susceptibility to infectious diseases [19][20]. TGF-β1 has a regulatory role in proliferation, differentiation, and activation of immune cells. Therefore, differences in its production or secretion in the individuals in a population might result in different responses to a given infectious agent [21][22]. An appropriate immune response to HCV infection is essential for its clearance from the blood and bodily fluids. Therefore, defective or insufficient production or secretion of cytokines could affect an individual's ability for virus clearance and prevention of chronic disease. As such, genetic variations, such as SNPs, in a population may affect an individual's susceptibility to chronic infectious diseases.The most commonly studied polymorphisms of TGF-β1 include a T/C transition at the -509 position of the promoter region, a T/C transition in codon 10, and a G/C transversion in codon 25. The genotypic distribution of TGF-β1 polymorphisms varies in different populations [23]. Several studies have investigated the relationship between polymorphisms in the TGF-β1 promoter or signal sequence and various diseases in the Iranian population. To our knowledge, however, there is limited information on the relationship between these polymorphisms and HCV infection in the Iranian population. Eurich et al. (2011) observed that the genotypes of TGF-β1 polymorphisms in codons 10 and 25 are significantly different in HCV patients with different degrees of hepatic fibrosis [24].Pereira et al. (2008) investigated the association of polymorphisms in TGF-β and three other cytokines with HCV infection in a Brazilian population. There was a statistically significant relationship between the polymorphism in codon 25 of TGF-β1 and HCV infection; however, there was no such association with the polymorphisms in codon 10 [3]. In contrast, Migita et al. observed that the site in codon 25 is not polymorphic among a Japanese population sample [25]; this finding was confirmed by Suzuki et al. [23]. A study in China by Xie et al. showed that all the 186 HBV patients in their study had the GG homozygous genotype in codon 25; they also found no significant difference in codon 10 or promoter region (-509) polymorphisms between the case and control groups [17]. These studies suggest that the site in codon 25 is not polymorphic among East Asian populations; this could be attributable to an ethnic difference between East Asians and other populations, such as Brazilians and Iranians. However, our results regarding codon 25 differ from those of previous studies. The polymorphism in codon 25 was detected in both the HCV and control groups; however, there was no significant difference in the frequency of the genotypes between the two groups. For the polymorphism in codon 10, our findings were consistent with those of a previous study conducted on a Brazilian population [3]; in both studies, there was no statistically significant difference in the genotype between the chronic HCV patients and healthy controls.Awad et al. [14] and Dunning et al. [15] observed that individuals with the G allele in TGF-β1 codon 25 exhibited a high cytokine-production phenotype. In our study, most of the Iranian patients in both the HCV and control groups exhibited high TGF-β1 production. Furthermore, according to Pereira et al. high TGF-β1 production is associated with increased HCV susceptibility; therefore, we hypothesize that patients with the GG genotype in codon 25 are more susceptible to HCV than other populations. Because of the predominance of a high-production phenotype among the studied Iranian population and the effect of high TGF-β levels on increased susceptibility to viral infections similar to HCV, we assume that most of the patients in our population are more susceptible than those in other populations, such as Brazilians. However, the results of our study indicate that TGF-β1 polymorphisms cannot be used as prognostic factors for chronic HCV infection in the Iranian population.

## References

[1] Shepard CW, Finelli L, Alter MJ (2005). Global epidemiology of hepatitis C virus infection. Lancet Infect Dis. Lancet Infect Dis.

[2] Szabo E, Lotz G, Paska C, Kiss A, Schaff Z (2003). Viral hepatitis: new data on hepatitis C infection. Pathol Oncol Res.

[3] Pereira FA, Pinheiro da Silva NN, Rodart IF, Carmo TM, Lemaire DC, Reis MG (2008). Association of TGF-beta1 codon 25 (G915C) polymorphism with hepatitis C virus infection. J Med Virol.

[4] Gerlach JT, Diepolder HM, Zachoval R, Gruener NH, Jung MC, Ulsenheimer A (2003). Acute hepatitis C: high rate of both spontaneous and treatment-induced viral clearance. Gastroenterology.

[5] Kondo Y, Ueno Y, Shimosegawa T (2011). Dysfunction of Immune Systems and Host Genetic Factors in Hepatitis C Virus Infection with Persistent Normal ALT. Hepat Res Treat.

[6] Koziel MJ (1999). Cytokines in viral hepatitis. Semin Liver Dis.

[7] Turner DM, Williams DM, Sankaran D, Lazarus M, Sinnott PJ, Hutchinson IV (1997). An investigation of polymorphism in the interleukin-10 gene promoter. Eur J Immunogenet.

[8] Clarke DC, Liu X (2008). Decoding the quantitative nature of TGF-beta/Smad signaling. Trends Cell Biol.

[9] Li MO, Sanjabi S, Flavell RA (2006). Transforming growth factor-beta controls development, homeostasis, and tolerance of T cells by regulatory T cell-dependent and -independent mechanisms. Immunity.

[10] Li MO, Sanjabi S, Flavell RA (2008). Transforming growth factor-beta (TGF-beta) and brain tumours. J Clin Neurosci.

[11] AT RV, Duarte MI, Pagliari C, Fernandes ER, Brasil RA, Benard G (2010). Tissue and serum immune response in chronic hepatitis C with mild histological lesions. Mem Inst Oswaldo Cruz.

[12] Lawrence DA (1996). Transforming growth factor-beta: a general review. Eur Cytokine Netw.

[13] Bedossa P, Paradis V (2003). Liver extracellular matrix in health and disease. J Pathol.

[14] Awad MR, El-Gamel A, Hasleton P, Turner DM, Sinnott PJ, Hutchinson IV (1998). Genotypic variation in the transforming growth factor-beta1 gene: association with transforming growth factor-beta1 production, fibrotic lung disease, and graft fibrosis after lung transplantation. Transplantation.

[15] Dunning AM, Ellis PD, McBride S, Kirschenlohr HL, Healey CS, Kemp PR, Luben RN, Chang-Claude J, Mannermaa A, Kataja V, Pharoah PD, Easton DF, Ponder BA, Metcalfe JC (2003). A transforming growth factorbeta1 signal peptide variant increases secretion in vitro and is associated with increased incidence of invasive breast cancer. Cancer Res.

[16] Cotton SA, Gbadegesin RA, Williams S, Brenchley PE, Webb NJ (2002). Role of TGF-beta1 in renal parenchymal scarring following childhood urinary tract infection. Kidney Int.

[17] Xie HY, Wang WL, Yao MY, Yu SF, Feng XN, Jin J, Jiang ZJ, Wu LM, Zheng SS (2008). Polymorphisms in cytokine genes and their association with acute rejection and recurrence of hepatitis B in Chinese liver transplant recipients. Arch Med Res.

[18] Wilson AG, Symons JA, McDowell TL, McDevitt HO, Duff GW (1997). Effects of a polymorphism in the human tumor necrosis factor alpha promoter on transcriptional activation. Proc Natl Acad Sci U S A.

[19] Gratchev A, Schledzewski K, Guillot P, Goerdt S (2001). Alternatively activated antigen-presenting cells: molecular repertoire, immune regulation, and healing. Skin Pharmacol Appl Skin Physiol.

[20] Murtaugh MP, Foss DL (2002). Inflammatory cytokines and antigen presenting cell activation. Vet Immunol Immunopathol.

[21] Moses HL, Arteaga CL, Alexandrow MG, Dagnino L, Kawabata M, Pierce DF Jr, Serra R (1994). TGF beta regulation of cell proliferation. Princess Takamatsu Symp.

[22] Reed SG (1999). TGF-beta in infections and infectious diseases. Microbes Infect.

[23] Suzuki S, Tanaka Y, Orito E, Sugauchi F, Hasegawa I, Sakurai M, Fujiwara K, Ohno T, Ueda R, Mizokami M (2003). Transforming growth factor-beta-1 genetic polymorphism in Japanese patients with chronic hepatitis C virus infection. J Gastroenterol Hepatol.

[24] Eurich D, Bahra M, Boas-Knoop S, Lock JF, Golembus J, Neuhaus R, Neuhaus P, Neumann UP (2011). Transforming growth factor beta1 polymorphisms and progression of graft fibrosis after liver transplantation for hepatitis C virus--induced liver disease. Liver Transpl.

[25] Migita K, Miyazoe S, Maeda Y, Daikoku M, Abiru S, Ueki T, Yano K, Nagaoka S, Matsumoto T, Nakao K, Hamasaki K, Yatsuhashi H, Ishibashi H, Eguchi K (2005). Cytokine gene polymorphisms in Japanese patients with hepatitis B virus infection--association between TGF-beta1 polymorphisms and hepatocellular carcinoma. J Hepatol.

